# The role of weekly nanoparticle albumin bound paclitaxel monotherapy as second line or later treatment for advanced NSCLC in China

**DOI:** 10.18632/oncotarget.21103

**Published:** 2017-09-20

**Authors:** Puyuan Xing, Yixiang Zhu, Ling Shan, Sipeng Chen, Xuezhi Hao, Junling Li

**Affiliations:** ^1^ National Cancer Center/Cancer Hospital, Chinese Academy of Medical Sciences and Peking Union Medical College, Beijing, China; ^2^ Department of Pathology, Fudan University Shanghai Cancer Center, Shanghai, China; ^3^ School of Public Health, Capital Medical University, Beijing, China

**Keywords:** advanced non-small cell lung cancer (NSCLC), multiple treatments, taxane, nanoparticle albumin bound paclitaxel (Nab-PTX), secreted protein acidic and rich in cysteine (SPARC)

## Abstract

For patients with pretreated advanced non-small cell lung cancer (NSCLC), more effective treatments are unmet. We conducted a study to explore the optimal treatment schedule of nanoparticle albumin bound paclitaxel (Nab-PTX) as a second line or later treatment for advanced NSCLC patients in China. Ninety-eight patients, who had experienced failure of prior treatment and received Nab-PTX monotherapy (130 mg/m^2^) on days 1, 8 of a 21-day cycle were included. The median progression-free survival (PFS) and overall survival (OS) were 4.34 months (95% confidence interval [CI] 3.508 to 5.165 months) and 11.73 months (95% CI 9.211 to 14.247 months), respectively. The objective responses rate (ORR) and disease control rate (DCR) were 22.4% and 74.5%. Prior treatment with taxane and line of therapy did not influence the efficacy of Nab-PTX. The main grade 3 to 4 toxicities were neutropenia (25.5%) and leukopenia (12.4%). Furthermore, 24 cases offered samples to assess secreted protein acidic and rich in cysteine (SPARC) expression. No statistical difference was observed in treatment efficacy between SPARC expression-negative and positive. The findings suggest that weekly Nab-PTX monotherapy is effective and well tolerated for patients with pretreated advanced NSCLC, regardless of prior taxane exposure or line of therapy.

## INTRODUCTION

Non-small cell lung cancer (NSCLC) is a subtype of lung cancer and patients with NSCLC are always at advanced stages when diagnosed [1]. Although new molecular agents, such as tyrosine kinase inhibitors (TKIs) targeting epidermal growth factor receptor (EGFR) or anaplastic lymphoma kinase (ALK) have improved the overall survival (OS) in patients with corresponding genetic alterations [2–3], and immune checkpoint inhibitors such as nivolumab or pembrolizumab demonstrated a significantly longer OS compared to cytotoxic agent (docetaxel) in previously treated patients with advanced NSCLC [4–5], the prognosis for advanced NSCLC patients remains relatively poor. For patients who experienced multiple treatment, such as EGFR/ALK-TKIs, solvent-based paclitaxel (Sb-PTX) and so on, an effective therapeutic regimen is still required.

Nanoparticle albumin bound paclitaxel (Nab-PTX) is an albumin-bound formulation, with nanoparticles of paclitaxel bound to human serum albumin. Due to the absence of solvent, this formulation can minimize the occurrence of hypersensitivity reactions [6]. Albumin has the natural ability to promote the delivery of cytotoxic drugs into tumors by initiating albumin receptor (gp60)-mediated transcytosis across endothelial cells and accumulate the drug in tumors cell by binding to secreted protein acidic and rich in cysteine (SPARC), thereby it can increase the local drug concentration and enhance the ability of tumor destruction [7–11]. Some studies showed that high stromal SPARC reactivity was correlated with poor prognosis in some tumors including NSCLC, pancreatic cancer, and ovarian cancer [12–14]. Unfortunately, the role of SPARC as a biomarker of response to Nab-PTX treatment for advanced NSCLC has not been determined yet.

Data from clinical trials have shown that Nab-PTX treatment for advanced NSCLC patients as first-line treatment was effective [15, 16]. In a phase III prospective open label clinical trial, 1,052 chemo-naive patients with stage IIIB/IV NSCLC were randomized to receive either Nab-PTX (100 mg/ m^2^ on days 1, 8, and 15) (*n* = 521) or Sb-PTX (200 mg/m^2^ on d1) (*n* = 531) combined with carboplatin on day 1 every 3 weeks. The study demonstrated that Nab-PTX group achieved higher objective response rate (ORR) (33% versus 25%, respectively; *p* = 0.005) and lower grade 3 to 4 neutropenia rate (47% versus 58%, respectively; *p* < 0.001) compared with Sb-PTX as first-line treatment [17]. While we found that Nab-PTX was most often prescribed as second-line or later treatment in clinic. Although a few studies [18–20] had reported that Nab-PTX as second line or later regimen of chemotherapy was effective in advanced NSCLC among Western populations and East Asia populations, and there was no significant difference, regardless of prior taxane treatment. While there has been no a standard usage of Nab-PTX for these patients. In an open-label, multicenter, single-arm phase II study, weekly Nab-PTX (100 mg/m^2^ on days 1, 8, and 15 of a 21-day cycle) for patients with previously treated advanced NSCLC (those who had received prior treatment with Sb-PTX were excluded), demonstrated promising activity and tolerant toxicity [21]. Additionally, previous study revealed that a single-agent Nab-PTX schedule of 260mg/m^2^ on day 1 of a 21-day cycle was also demonstrated a favorable tolerability of the regime [20]. However, we found that patients with Nab-PTX (260mg/m^2^ on day 1 of a 21-day cycle) treatment mostly needed dose reduction or delayed treatment, due to adverse events in clinic, and Nab-PTX (100 mg/m^2^ on days 1, 8, and 15 of a 21-day cycle) regimen would increase patient’ hospitalization days for Nab-PTX (100 mg/m^2^ on days 1, 8, and 15 of a 21-day cycle). We performed this study to explore the optimal treatment schedule of Nab-PTX for Chinese advanced NSCLC patients who had experienced failure of prior treatment, and elucidate the potential influence of prior taxane treatment and SPARC expression on its efficacy.

## RESULTS

### Patient demographics

A total of 98 patients were enrolled in this study. Sixty-one patients had been previous treated with taxane, and 37 patients hadn’t. The median follow-up period was 21.56 months (range, 0.92 to 46 months). The primary progression-free survival (PFS) and OS analyses clinical cutoffs (July 31, 2015) were triggered by the 97th PFS event and 73rd OS event. The patient characteristics were listed in Table 1. All patients were with Eastern Cooperative Oncology Group performance status (ECOG PS) ≤ 2. The median age was 61 years old for patients with prior taxane treatment (range, 41 to 81 years), and 62 years old for patients without prior taxane treatment (range, 35 to 80 years). Forty-one patients (67.2%) were male for patients with prior taxane treatment, while 29 patients (78.6%) were male for patients without prior taxane treatment. The median of number of treatment cycles was 4 for patients with prior taxane treatment (range, 2 to 10 cycles), as well as those without prior taxane treatment (range, 2 to 12 cycles). Median previous treatment line was 3 for patients with prior taxane treatment (range, 1 to 8 lines). Median previous treatment line was 2 for patients without prior taxane treatment (range, 1 to 4 lines). Baseline characteristic variables were balanced with no statistical difference between groups with and without prior taxane-based therapy, except for stage (*p* = 0.001) and the previous treatment line (*p* < 0.001).

**Table 1:** Baseline characteristics of all patients

Characteristics	Total, *n* (%)	Prior Taxane		*p*
		Yes, *n* (%)	No, *n* (%)	
**Age**				0.59
< 60	41 (41.8)	23 (37.7)	18 (48.7)	
60 ≤ age < 70	38 (38.8)	29 (47.5)	9 (24.3)	
≥ 70	19 (19.4)	9 (14.8)	10 (27.0)	
**Gender**				
Male	70 (71.4)	41 (67.2)	29 (78.6)	0.236
Female	28 (28.6)	20 (32.8)	8 (21.4)	
**ECOG performance status**				
0	11 (11.2)	7 (11.5)	4 (10.8)	0.292
1	83 (84.7)	53 (81.9)	30 (81.1)	
2	4 (4.1)	1 (1.6)	3 (8.1)	
**Smoking**				
No	46 (46.9)	31 (50.8)	15 (40.5)	0.323
Yes	52 (53.1)	30 (49.2)	22 (59.5)	
**Clinic stage**				
IIIB	5 (5.1)	0	5 (13.5)	0.001
IV	76 (77.6)	54 (88.5)	22 (59.5)	
Postoperative recurrence	17 (17.3)	7 (11.5)	10 (27.0)	
**Pathological type**				
Adenocarcinoma	66 (67.3)	44 (72.1)	22 (59.5)	0.429
Squamous carcinoma	30 (30.6)	16 (26.2)	14 (37.8)	
Other	2 (2.1)	1 (1.6)	1 (2.7)	
**Number of treatment cycles**				0.083
< 4	37 (37.8)	28 (45.9)	9 (24.3)	
4 or 5	31 (31.6)	18 (29.5)	13 (35.1)	
≥ 6	30 (30.6)	15 (24.6)	15 (40.6)	
**Prior line of therapy**				< 0.001
1st line	16 (16.3)	3 (4.9)	13 (35.2)	
2nd line	26 (26.5)	16 (26.2)	10 (27.0)	
≥ 3rd line	56 (57.2)	42 (68.9)	14 (37.8)	
**EGFR/ALK- mutation status**				
Wild type	35 (35.7)	23 (37.7)	12 (32.4)	0.867
Mutant type	18 (18.4)	11 (18.0)	7 (18.9)	
Unknown	45 (45.9)	27 (44.3)	18 (48.7)	
**Prior EGFR/ALK-TKIs**				
No	33 (33.7)	20 (32.8)	13 (35.1)	0.812
Yes	65 (66.3)	41 (67.2)	24 (64.9)	
**Prior lung radiotherapy**				
No	57 (58.2)	35 (57.4)	22 (59.5)	0.839
Yes	41 (41.8)	26 (42.6)	15 (40.5)	

n, number; Prior taxane, prior taxane treatment; p, p value; ECOG, Eastern Cooperative Oncology Group; EGFR, epidermal growth factor receptor; ALK, anaplastic lymphoma kinase; TKIs, tyrosine kinase inhibitors.

### Survival and response

**Figure 1:**
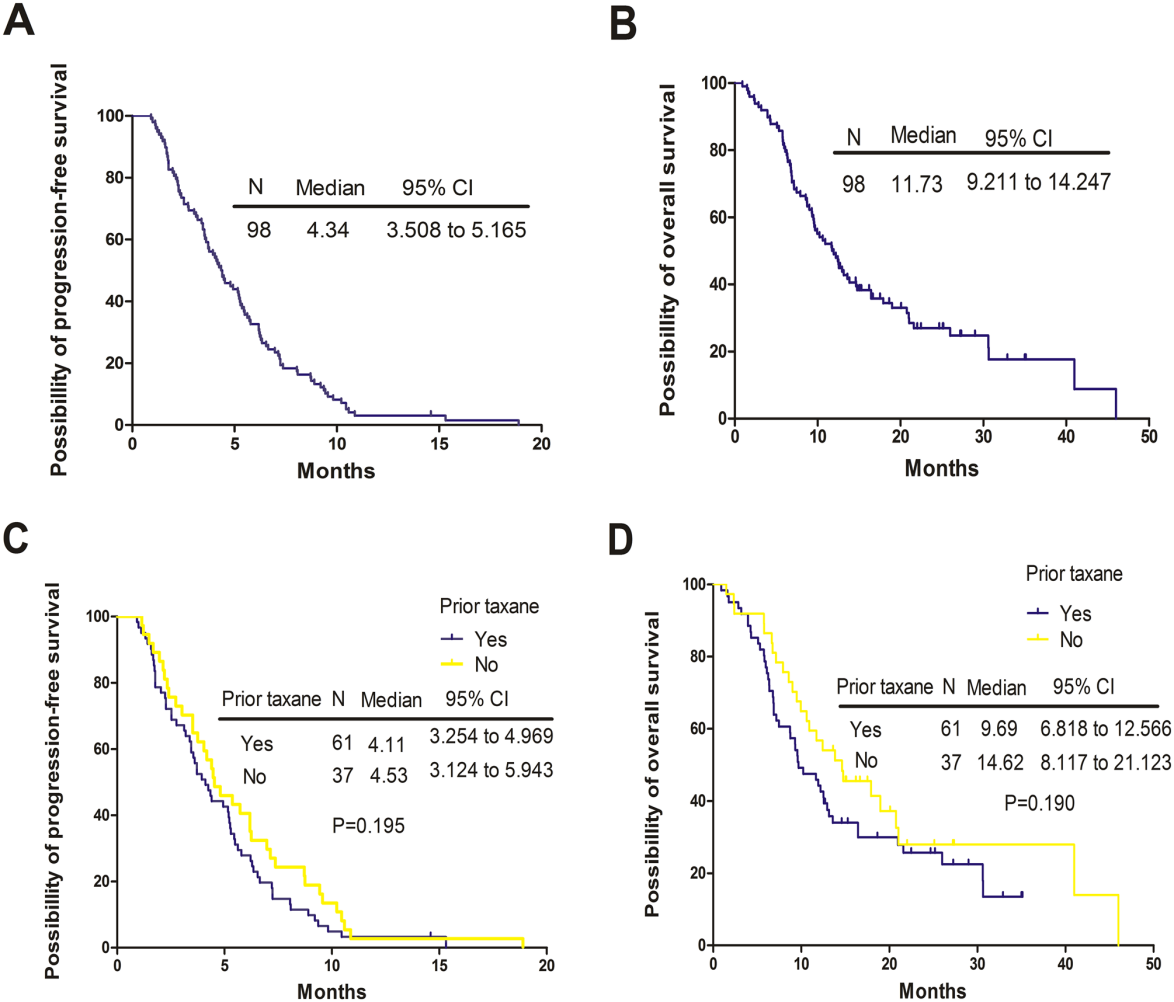
Kaplan-Meier curves for progression-free survival (PFS) and overall survival (OS) for the entire populations and patients with or without prior taxane treatment (**A**) The PFS curves for all patients. (**B**) The OS curves for all patients. (**C**) The PFS curves stratified by prior taxane exposure. (**D**) The OS curves stratified by prior taxane exposure. N, number; CI, confidence interval; *p*, *p* value.

The median PFS and OS for all patients were 4.34 months (95% confidence interval [CI]: 3.508 to 5.165 months) and 11.73 months (95% CI 9.211 to 14.247 months), respectively. The median PFS was 4.11 months for patients with prior taxane treatment (95% CI 3.254 to 4.969 months), versus 4.53 months (95% CI 3.124 to 5.943 months) for patients without prior taxane treatment (*p* = 0.195). The median OS was 9.69 months for patients with prior taxane treatment (95% CI 6.818 to 12.566 months) versus 14.62 months for patients without prior taxane treatment (95% CI 8.117 to 21.123 months). In spite of the favorable OS trend in the patients who did not receive prior taxane-based therapy, statistical significance was not observed (*p* = 0.190) (Figure 1). All patients were evaluated for drug efficacy. The ORR and disease control rate (DCR) of all patients were 22.4% and 74.5% (0 complete remission [CR], 22 partial response [PR], and 51 stable disease [SD]), respectively (Table 2). The ORR and DCR were 23.0% and 70.5% (0 CR, 14 PR, and 29 SD) for patients with prior taxane treatment and 21.6% and 81.1% (0 CR, 8 PR, and 22 SD) for patients without prior taxane treatment (Table 3). ORR (*p* = 0.533) and DCR (*p* = 0.244) had no significant differences between the two groups (Table 3).

**Table 3:** Treatment outcome according to prior taxane exposure

Overall best response	Prior Taxane		
	Yes, *n* (%)	No, *n* (%)	*p*
CR	0	0	
PR	14 (23.0)	8 (21.6)	
SD	29 (47.5)	22 (59.5)	
PD	18 (29.5)	7 (18.9)	
ORR	14 (23.0)	8 (21.6)	0.533
DCR	43 (70.5)	30 (81.1)	0.244

Prior taxane, prior taxane treatment; n, number; p, *p* value; CR, complete remission; PR, partial response; SD, stable disease; PD, disease progression; ORR, objective responses rate (ORR = CR + PR); DCR, disease control rate (DCR = CR + PR + SD).

**Table 2:** Treatment outcome of all patients

Overall best response	*n* (%)
CR	0
PR	22 (22.4)
SD	51 (52.1)
PD	25 (25.5)
ORR	22 (22.4)
DCR	73 (74.5)

n, number; CR, complete remission; PR, partial response; SD, stable disease; PD, disease progression; ORR, objective responses rate (ORR = CR + PR); DCR, disease control rate (DCR = CR + PR + SD).

### Safety and tolerability

All patients who received weekly Nab-PTX monotherapy treatment were eligible for safety analysis. All-grade treatment-related adverse events (AEs) were shown in Table 4. The most frequent treatment-related toxicities were mild or moderate (grades 1 or 2). Hematologic adverse toxicities were the main grade 3 to 4 toxicities, including neutropenia (25.5%), leukopenia (12.4%) and anemia (3.1%). The main grade 3 to 4 nonhematologic AEs were peripheral neuropathy (5.1%), myalgia/arthralgia (5.1%) and fatigue (1.0%), which were lower than hematologic adverse toxicities (Table 3). There were no treatment-related deaths.

**Table 4:** Adverse events of all patients

Adverse events	Grades 0,*n* (%)	Grades 1,*n* (%)	Grades 2,*n* (%)	Grades 3,*n* (%)	Grades 4,*n* (%)
Hematologic adverse events Anemia	61 (62.2)	24 (24.5)	10 (10.2)	3 (3.1)	
Leukopenia	38 (38.7)	16 (16.3)	32 (32.6)	12 (12.4)	
Neutropenia	22(22.5)	17 (17.3)	34 (34.7)	16 (16.3)	9 (9.2)
Thrombocytopenia	87 (88.8)	6 (6.1)	5 (5.1)		
Nonhematologic adverse events					
Nausea and vomiting	56 (57.1)	35 (35.7)	7 (7.2)		
Alopecia	69(70.4)	27 (27.6)	2 (2.0)		
Diarrhea	96 (98.0)	1 (1.0)	1 (1.0)		
Mucositis	89(90.8)	9 (9.2)			
Fatigue	74 (75.5)	16 (16.3)	7 (7.2)	1 (1.0)	
peripheral neuropathy	51 (52.0)	29 (29.6)	13 (13.3)	5 (5.1)	
Myalgia/Arthralgia	59 (60.2)	29 (29.6)	5 (5.1)	5 (5.1)	
ALT/AST elevation	84 (85.7)	12 (12.3)	2 (2.0)		

n, number; ALT, alanine transaminase; AST, aspartate transaminase.

### Risk factors for PFS according to Cox proportional hazards regression model Cox proportional hazards regression model (COX) analysis

Univariate analyses showed that compared with PS 0, patients with PS 1 might have a better PFS (hazard ratio [HR] 0.521, 95% CI 0.275 to 0.988, *p* = 0.046). Compared with therapy cycles less than 4, 4 to 5 cycles (HR 0.256, 95% CI 0.155 to 0.424, *p* < 0.001) and cycles more than and equal to 6 (HR 0.144, 95% CI 0.084 to 0.248, *p* < 0.001) were associated with a superior survival. In multivariate analyses, we included PS, stage, age, pathological type, the number of previous therapy lines, the number of treatment cycles, prior taxane treatment status, EGFR or ALK mutation status, prior EGFR/ALK-TKIs status and prior lung radiotherapy status. The data also revealed that patients with PS 1 had longer PFS than those with PS 0 (HR 0.475, 95% CI 0.247 to 0.915, *p* = 0.026), and 4 to 5 cycles (HR 0.230, 95% CI 0.135 to 0.394, *p* < 0.001) and cycles more than and equal to 6 (HR 0.107, 95% CI 0.059 to 0.193, *p* < 0.001) were associated with a superior survival compared with therapy cycles less than 4 (Table 5). In addition, compared patients who did not receive prior EGFR/ALK-TKIs, those receiving prior EGFR/ALK-TKIs had an inferior survival (HR 2.101, 95% CI 1.245 to 2.604, *p* = 0.005).

**Table 5:** Univariate and multivariate analysis in Cox proportional hazards regression model

Characteristics	Univariate analysis				Multivariate analysis			
	*p*	HR	95% CI		*p*	HR	95% CI	
**ECOG performance status**								
1 versus 0	0.046	0.521	0.275	0.988	0.026	0.475	0.247	0.915
2 versus 0	0.851	0.896	0.284	2.830	0.408	0.567	0.148	2.173
**Smoking**								
Yes versus No	0.550	0.885	0.592	1.323				
**Stage**								
**IV versus IIIB**	0.946	0.969	0.390	2.405	0.939	0.962	0.351	2.637
Postoperative recurrence versus IIIB	0.885	0.929	0.342	2.523	0.746	1.205	0.389	3.738
**Gender**								
Female versus male	0.711	1.088	0.697	1.699				
**Age**								
< 70 versus < 60	0.393	0.822	0.524	1.290	0.341	0.799	0.503	1.269
≥ 70 versus < 60	0.273	0.731	0.418	1.280	0.544	0.827	0.449	1.526
**Pathological type**								
Squamous versus Adenocarcinoma	0.458	0.844	0.540	1.321	.782	1.088	0.599	1.975
Unknown versus Adenocarcinoma	0.343	1.986	0.481	8.199	0.560	1.541	0.360	6.603
**EGFR/ALK-mutation status**								
Mutant versus Wild type	0.473	0.811	0.457	1.438	0.499	1.280	0.625	2.621
Unknown versus Wild type	0.752	0.930	0.595	1.456	0.435	1.829	0.403	8.321
**Prior taxane treatment**								
Yes versus No	0.198	1.314	0.867	1.993	0.353	0.796	0.210	2.383
**Prior EGFR/ALK-TKIs**								
Yes versus No	0.555	1.138	0.740	1.751	0.005	2.101	1.245	2.604
Prior line of therapy								
2nd versus 1st	0.164	1.605	0.824	3.124	0.930	0.961	0.395	2.338
≥ 3rd versus 1st	0.180	1.499	.830	2.706	0.780	1.162	0.406	3.326
**Number of treatment cycles**								
4 or 5 versus < 4	< .001	0.256	0.155	0.424	< .001	0.230	0.135	0.394
≥ 6 versus < 4	< .001	0.144	0.084	0.248	< .001	0.107	0.059	0.193
**Prior lung radiotherapy**								
Yes versus No	0.209	1.301	0.863	1.962	0.089	1.520	0.938	2.465

*p*, *p* value; HR, hazard ratio; CI, confidence interval; ECOG, Eastern Cooperative Oncology Group; EGFR, epidermal growth factor receptor; ALK, anaplastic lymphoma kinase; TKIs, tyrosine kinase inhibitors; Prior taxane, prior taxane treatment.

### Efficacy in accordance with biomolecular expression

**Figure 2:**
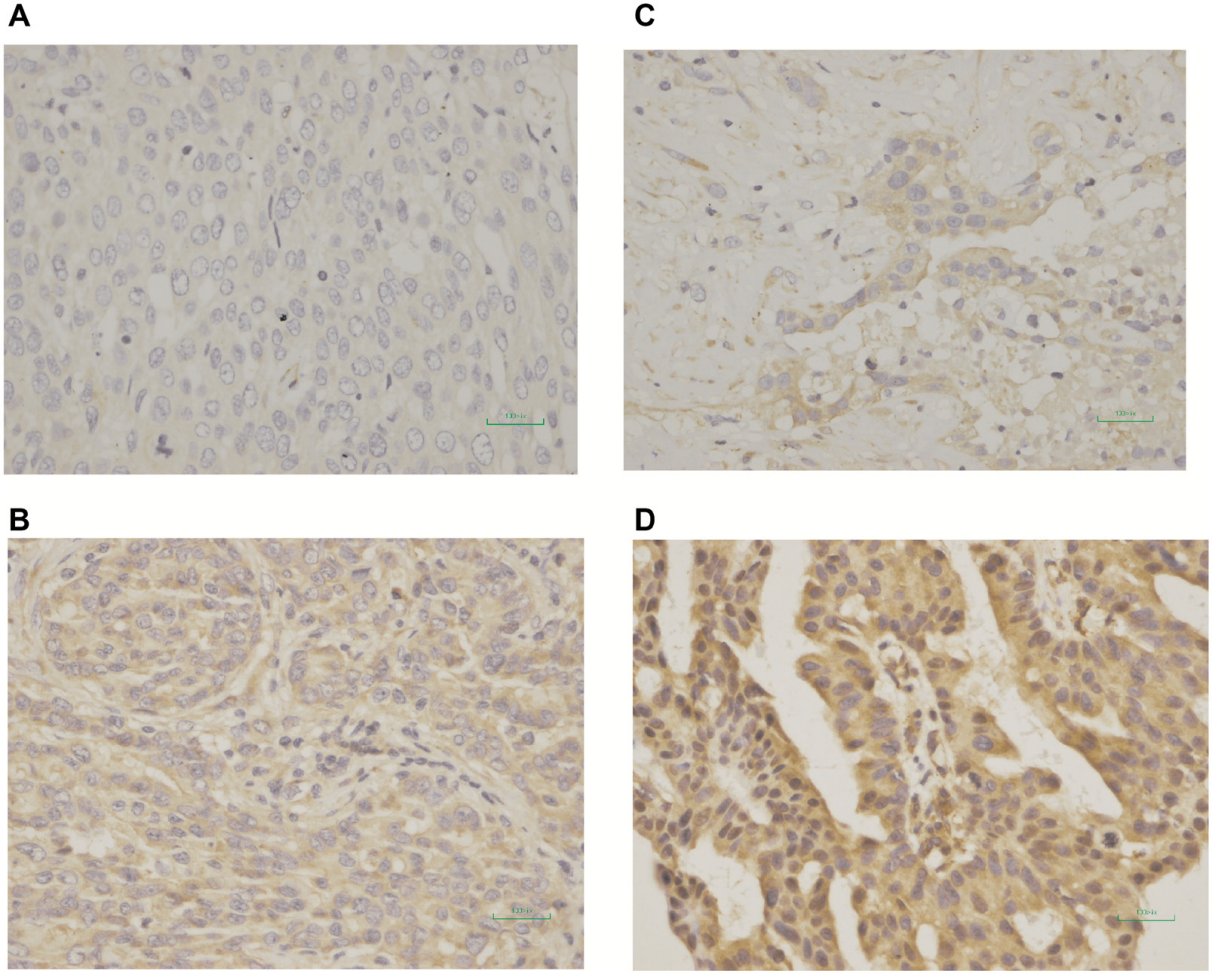
Immunohistochemical expression using monoclonal antibody (R&D system, MAB941) of secreted protein acidic and rich in cysteine (SPARC) (**A**) Tumors with score of 0; (**B**) Tumors with score of 1+; (**C**) Tumors with score of 2+; (**D**) Tumors with score of 3+. Tumors with a score of 0 or 1+ were regarded as negative for SPARC expression, and those with a score of 2+ or 3+ were regarded as positive for SPARC expression.

**Figure 3:**
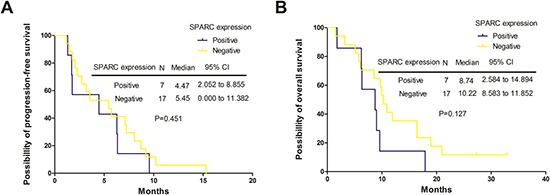
The Progression-free survival (PFS) and overall survival (OS) curves stratified by secreted protein acidic and rich in cysteine (SPARC) expression (**A**) The PFS curves stratified by SPARC expression. (**B**) The OS curves stratified by SPARC expression. Negative, tumors with negative SPARC expression; Positive, tumors with positive SPARC expression; N, number; CI, confidence interval; p, *p* value.

A total of 24 cases (15 patients with prior taxane treatment and 9 patients without prior taxane treatment) offered samples to assess SPARC expression. Seventeen cases (17.3%) were negative for SPARC expression (−/1+) and 7 cases (7.1%) were positive (2+/3+) (Figure 2). Tables 4 demonstrated results regarding efficacy, and Figure 3 showed survival according to biomolecular expression. ORR was 35.5% for the SPARC expression-negative group and 14.2% for SPARC expression-positive group. DCR was 71.0% for the SPARC expression-negative group and 57.1% for SPARC expression-positive group. There were no statistically significant differences in ORR (*p* = 0.303) and DCR (*p* = 0.525) between the SPARC expression-negative and SPARC expression-positive group (Table 6). The median PFS was 5.45 months (95% CI 2.052 to 8.855 months) versus 4.47 months (95% CI 0.000 to 11.382 months) for SPARC expression negative and positive group (*p* = 0.451). The median OS was 10.22 months (95% CI 8.583 to 11.852 months) versus 8.74 months (95% CI 2.584 to 14.894 months) for for SPARC expression negative and positive group (*p* = 0.127) (Figure 3).

**Table 6:** Treatment outcome according to secreted protein acidic and rich in cysteine (SPARC) expression

Overall best response	SPARC expression		
	Negative, *n* (%)	Positive, *n* (%)	*p*
CR	0	0	
PR	6 (35.5)	1 (14.2)	
SD	6 (35.5)	3 (42.9)	
PD	5 (29.0)	3 (42.9)	
ORR	6 (35.5)	1 (14.2)	0.303
DCR	12 (71.0)	4 (57.1)	0.525

n, number; *p*, *p*-value; CR, complete remission; PR, partial response; SD, stable disease; PD, disease progression; ORR, objective responses rate (ORR = CR + PR); DCR, disease control rate (DCR = CR + PR + SD).

## DISCUSSION

Docetaxel as second-line therapy for advanced NSCLC improved survival by a median of 3.0 months with a response rate less than 10% [22, 23]. Erlotinib in second or third line setting had a median PFS of 2.2 months with a response rate of 8.9% [24]. Anti-programmed death 1 (anti-PD-1) antibodies nivolumab and pembrolizumab as second or later treatment obtained median PFS ranging from 2.3 to 3.7 months among populations including both programmed death ligand 1 (PD-L1) positive and negative tumors [4, 25]. Our analyses displayed an ORR of 22.4%, a median PFS and OS of 4.34 months and 11.73 months in Nab-PTX treatment for advanced NSCLC patients who experienced failure of prior treatment, which suggested that Nab-PTX might possess convincing antitumor activity compared with those previously reported second line therapies [4, 23–25].

In a phase II study, Nab-PTX (100 mg/ m^2^ on days 1, 8, 15 of a 28-day cycle) as second-line treatment for Chinese patients with advanced NSCLC (*n* = 56) yielded an ORR of 16.1% (95% CI 8.9% to 24.7%) and PFS of 3.5 months (95% CI 1.9 to 5.8 months), respectively [26]. A phase II trial from Japan adopted Nab-PTX (100 mg/m^2^ on days 1, 8, and 15 of a 21-day cycle) for platinum-refractory advanced NSCLC patients (*n* = 41) (patients with prior Sb-PTX treatment were excluded). The outcomes showed a median PFS and OS of 4.9 months (95% CI 2.4 to 7.4 months) and 13.0 months (95% CI 8.0 to 18.0 months), respectively [21]. Another report from Western populations indicated that Nab-PTX (260 mg/m^2^ on d1 of a 21-day cycle) in patients with relapsed or platinum-refractory advanced NSCLC (*n* = 31) had an ORR of 16%, a DCR of 64.5%, and a median treatment failure-free survival (TFFS) of 3.5 months for patients who received Nab-PTX in second-line or later treatment [18]. In the present study, we enrolled 98 advanced NSCLC patients who had experienced failure of prior treatment, and adopted a single-agent Nab-PTX schedule of 130 mg/m^2^ on days 1, 8 of a 21-day cycle. Our analyses, in line with above studies, showed that weekly Nab-PTX monotherapy was effective in the second line and later treatment, which suggested that Eastern and Western populations might equally benefit from the drug.

In clinical practice, Nab-PTX is common to be subsequent treatment regimen for advanced NSCLC patients after Sb-PTX or docetaxel treatment failure. However, whether prior taxane exposure and line of therapy were correlated with the efficacy of Nab-PTX therapy remained unclear. In our study, the data indicated that Nab-PTX was most often prescribed as third-line or later treatment, and most patients had been previously treated with taxane. The study among Western populations showed that there were no statistically significant difference in TFFS with prior taxane exposure (median TFFS of 3.5 months for patients without prior taxane versus 2.2 months for patients with prior taxane; *p* = 0.10) as well as line of therapy (median TFFS of 2.2 months for second-line treatment versus. 3.6 months for ≥ third-line treatment; *p* = 0.78) [18]. The analysis in Chinese populations demonstrated that weekly administering Nab-PTX monotherapy (100 mg/m^2^ on days 1, 8, and 15 of a 28-day cycle) as a second-line chemotherapy in elderly patients with squamous NSCLC was an effective and safe regimen for relapsed NSCLC in terms of ORR, DCR, PFS, and OS, regardless of prior taxane treatment according to the subgroup analysis (*p* = 0.952) [20]. The median PFS in our study for patients with prior taxane treatment was similar to those without prior taxane treatment (median, 4.11 versus 4.54 months, *p* = 0.195). The results of median OS were 9.69 months for patients with prior taxane treatment and 14.62 months for patients without prior taxane treatment. What is noteworthy is that although the difference in OS was not statistically significant for patients regardless of prior taxane exposure (*p* = 0.190), the median OS of those without prior taxane treatment were almost prolonged by 3.0 months. We looked forward to multicenter randomized controlled trials with larger sample size to confirm it. Our analysis revealed that the efficacy of Nab-PTX as earlier line treatment had no superior PFS than later line of therapy (3rd line versus 2nd line, HR 1.605, 95% CI 0.824 to 3.124, *p* = 0.164; (3rd line versus 2nd line, HR 1.499, 95% CI 0.830 to 2.706, *p* = 0.180). The results in both the univariate and multivariate analyses were consistent with above ones, which suggested that weekly Nab-PTX monotherapy remained effective regardless of prior taxane exposure and line of therapy.

The main grade 3 to 4 toxicities observed in our study included neutropenia (25.5%), followed by leukopenia (12.4%), peripheral neuropathy (5.1%) and myalgia/arthralgia (5.1%), anemia (3.1%), and fatigue (1.0%). Similar results were found in other reports, showing a favorable toxicity profile for Nab-PTX monotherapy regimen [18, 26, 27]. In a clinical trial with larger patient sample, the incidence of grade 3 to 4 neutropenia in a schedule of Nab-PTX (100 mg/m^2^ on days 1, 8 and 15 of a 21-day cycle) plus carboplatin administered every 21 days was 47% [17]. In previous studies, Nab-PTX combined with carboplatin or nedaplatin regimen revealed a higher incidence of grade 3 to 4 hematologic toxicities (62.5% to 80.0%) [28–30]. Compared with the above results [17, 28–30], weekly Nab-PTX alone (130 mg/m^2^ on days 1, 8 of a 21-day cycle) in our study was beneficial for advanced NSCLC patients with better tolerability and fewer AEs. Therefore, weekly Nab-PTX monotherapy (130 mg/m^2^ on days 1, 8 of a 21-day cycle) regimen displayed promising safety and feasibility profiles for previously treated patients with advanced NSCLC patients.

In the COX analysis, the PFS for patients receiving fewer than four cycles of treatment was worse than that of those receiving more than four cycles of treatment (*p* < 0.001). Several meta-analyses indicated patients with more cycles of chemotherapy had significant longer PFS than those with fewer cycles of treatment [31–33]. Maybe we can advocate patients to receive more cycles of treatment until disease progression or occurrence of unacceptable toxicity. Unfortunately, we failed to explore whether the result was consistent with OS. Furthermore, we found that patients with PS 1 and without previous EGFR/ALK-TKIs had a superior PFS than those with PS 0 (HR 0.475, 95% CI 0.247 to 0.915, *p* = 0.026), and previous EGFR/ALK-TKIs (HR 2.101, 95% CI 1.245 to 2.604, *p* = 0.005). A possible contributing reason for former result might be that 84.7% of patients in our study were PS 1, which was far more than patients with PS 0, leading to the bias of our data. Previous study had reported that PS was not a potential factor that influenced the efficacy of Nab-PTX [34]. As for the latter, the reason remained unclear. However, we found that PFS of patients with previous EGFR/ALK-TKIs were equal to those prior without TKIs in univariate analysis. While the result was contrary in multivariate analysis. Hence, we proposed one possible reason was due to the interaction of the enrolled variables. Another explanation was that previous EGFR/ALK-TKIs was an independent factor for PFS. However, most of patients with EGFR mutation–negative or unknown mutation status treated with EGFR/ALK-TKIs in our study. Unfortunately, the survival benefit was not driven by them [35], which leaded to a negative result in univariate analysis. When multivariate analysis was used, some potential confounding factors were adjusted, resulting in previous EGFR/ALK-TKIs treatment becoming an independent risk factors for survival. We are looking forward to more studies to confirm its reliability.

Whether SPARC expression was a predictive biomarker to Nab-PTX therapy remains controversial. Some literatures [36, 37] found that high stromal SPARC expression in the primary tumor might be a biomarker for Nab-PTX treatment of inferior PFS and OS in advanced pancreatic ductal adenocarcinoma and better survival in NSCLC. However, another study reported that SPARC expression did not seem to be associated with the efficacy of Nab-PTX in metastatic breast cancer [38]. In our study, the result revealed that there was no statistically significant difference between the SPARC expression-negative or positive group in ORR (*p* = 0.303) and DCR (*p* = 0.525). Moreover, no difference of PFS (*p* = 0.451) and OS (*p* = 0.127) was observed in the SPARC expression-negative or positive group. The reason might be due to the selection bias caused by low percentage of SPARC expression tests. In addition, higher SPARC expression used to be found in squamous cell carcinoma [37]. However, in this study, there was a higher proportion of adenocarcinoma than squamous cell lung cancer, which perhaps resulted in no significant correlation between SPARC expression and the curative effect of Nab-PTX.

Indeed, our work had several limitations. First, this study is a retrospective analysis with a small sample size. A prospective, larger sample, multicenter clinical study is needed to verify these results. Moreover, as a single-center and small sample study, patients who received Nab-PTX treatment might be selected. Last but not least, previous chemotherapy schemes and the dosage were not included in our analysis.

In summary, weekly Nab-PTX monotherapy schedule of 130 mg/m^2^ on days 1 and 8 of a 21-day cycle was efficacious and well-tolerated for patients with pretreated advanced NSCLC in Chinese population, regardless of line of therapy, prior taxane exposure or SPARC expression. Continuation maintenance with Nab-PTX monotherapy could prolong PFS for them. Consequently, weekly Nab-PTX monotherapy would be a favorable option for these patients, especially for those failed in multiple treatment. Further randomized controlled trials with large sample size are needed to explore Nab-PTX in the treatment of recurrent advanced NSCLC.

## MATERIALS AND METHODS

### Study design

The study was approved by the Ethics Committee of the Cancer Hospital of the Chinese Academy of Medical Sciences (CAMS) (Beijing, China) (approval number: 15-079/1006). From June 2010 to July 2015, 98 advanced NSCLC patients who had been treated with weekly Nab-PTX monotherapy with advanced NSCLC were included in this study. Patient data included PFS and OS, age, gender, ECOG PS, smoking history, stage, pathological type, number of previous treatment lines, number of treatment cycles of Nab-PTX, prior taxane treatment status, EGFR/ALK-mutation status, prior EGFR/ALK-TKIs treatment status, previous lung radiotherapy status, treatment response, and toxicities. PFS was assessed by investigators with computed tomography (CT) scan, magnetic resonance imaging (MRI), bone scanning, and tumor markers. All staging procedures were carried out using the 7th Union for International Cancer Control tumor node metastasis (TNM) classification. PS was defined according to the ECOG performance scale [39]. The primary end point was PFS. Secondary end points included ORR, OS, and safety. Exploratory analyses for SPARC expression status in tumor tissues were performed as well. SPARC expression status was tested by immunohistochemistry using monoclonal antibody (R&D system, MAB941). Tumors with a score of 0 or 1+ were regarded as negative for SPARC expression, and those with a score of 2+ or 3+ were regarded as positive for SPARC expression [40]. By the end of July 31, 2015, data obtained from multiple sources including clinical letters, follow-up scans, hospital computer information systems and telephone follow-ups were extracted into our database for analyses.

### Patient selection

Advanced NSCLC patients who had been treated with weekly Nab-PTX monotherapy in the CAMS were included in this study. Those who had previously received at least one line of systemic therapy (either chemotherapy or TKIs) and received Nab-PTX in later regimen were also included.

### Treatment

Nab-PTX (Abraxane^®^, Abraxis, USA; 100 mg/vial) was given at a dose of 130 mg/m^2^ over 30 minutes’ infusion on days 1 and 8 of a 21-day cycle. Patients were scheduled to receive at least 2 cycles and the therapeutic efficacy was evaluated after every two cycles. It was allowed to evaluate treatment efficacy when patients occured symptom aggravating after 1 cycle.

### Efficacy and safety

The Response Evaluation Criteria in Solid Tumors (RECIST) 1.1 criteria was used for efficacy evaluation in terms of CR, PR, SD, PD, confirmed complete and partial responses (ORR = CR+PR) and DCR (DCR = CR+PR+SD) [41]. Any adverse medical event that happened between the initiation and one month after completion of the investigational treatment was recorded as AEs, regardless of whether the AEs were associated with the drug. The evaluation of AEs was based on the National Cancer Institute-Common Toxicity Criteria (NCI-CTC) 3.0 version. PFS was measured as the duration from start the treatment to recurrence (local, regional, and/or distant), measured by CT scans, MRI, bone scanning, or positron emission tomography (PET)/CT scans, or death from any cause before recurrence. OS was defined as the duration from the start of the treatment to death of any cause [42].

### Statistical analysis

The patients’ characteristics and responses were analyzed using descriptive methods. Continuous variables were compared using *t* tests, and categorical variables were compared using χ2 tests. PFS and OS were calculated with Kaplan-Meier product limit method. The numbers and incidences of AEs were summarized using descriptive statistics, absolute frequencies, and percentages in the tables. The Cox proportional hazard regression model was used to identify risk factors independently associated with PFS. In univariate analysis, all variables including age, gender, PS, smoking history, stage, pathological type, number of previous treatment lines, number of treatment cycles, prior taxane treatment status, EGFR/ALK-mutation status, prior receiving EGFR/ALK-TKIs treatment status, and previous lung radiotherapy status were performed. In multivariate analysis, the factors which were significantly associated with PFS by univariate analysis and might influence the Nab-PTX therapy in previous studies were included. All statistical analyses were performed using SPSS version 17 and a *p value* < 0.05 was considered significant.

## Abbreviations

NSCLC: non-small cell lung cancer
Nab-PTX: nanoparticle albumin bound paclitaxel
PFS: progression-free survival
OS: overall survival
CI: confidence interval
HR: hazard ratio
ORR: objective responses rate
DCR: disease control rate
CR: complete remission
PR: partial response
SD: stable disease
PD: disease progression
SPARC: secreted protein acidic and rich in cysteine
TKIs: tyrosine kinase inhibitors
EGFR: epidermal growth factor receptor
ALK: anaplastic lymphoma kinase
ECOG PS: Eastern Cooperative Oncology Group performance status
AEs: adverse events
anti-PD-1: anti-programmed death-ligand 1
anti-PD-1: anti-programmed death 1
PD–L1: programmed death ligand 1
TFFS: treatment failure-free survival
COX: Cox proportional hazards regression model Cox proportional hazards regression model
CAMS: Cancer Hospital of the Chinese Academy of Medical Sciences
CT: computed tomography
MRI: magnetic resonance imaging
PET: positron emission tomography
TNM: tumor node metastasis
RECIST: Response Evaluation Criteria in Solid Tumors
NCI-CTC: National Cancer Institute-Common Toxicity Criteria.
